# Cord-Based Microfluidic Chips as A Platform for ELISA and Glucose Assays

**DOI:** 10.3390/mi10090614

**Published:** 2019-09-15

**Authors:** Jenny Elomaa, Laura Gallegos, Frank A. Gomez

**Affiliations:** Department of Chemistry and Biochemistry, California State University, Los Angeles, 5151 State University Drive, Los Angeles, CA 90032-8202, USA; jelomaa@calstatela.edu (J.E.); laura_g13@live.com (L.G.)

**Keywords:** enzyme-linked immunosorbent assay, microfluidics, microfluidics cord-based analytical device, point-of-care diagnostic device

## Abstract

This paper describes the development and application of microfluidic cord-based analytical devices (µCADs) in two enzyme-linked immunosorbent assays (ELISAs) and glucose assay. In this study, biotinylated goat anti-mouse immunoglobulin (IgG) antibody, rabbit IgG antibody, and glucose are quantitatively detected. In the ELISA systems, the antibody is spotted on the cord at the detection site and a series of washes, followed by streptavidin-alkaline phosphatase (Strep-ALP) or alkaline phosphatase (ALP)-conjugated secondary antibody and colorimetric substrate, completing the experiment. The devices are subsequently scanned and analyzed yielding a correlation between inverse yellow or inverse blue intensity and antibody concentration. For the first ELISA, a linear range of detection was observed at lower concentrations (2.50 × 10^−4^–1.75 × 10^−3^ mg/mL) of Strep-ALP with saturation of the enzyme achieved at higher concentrations (>2.50 × 10^−4^). For the second ELISA, the L_50_ was demonstrated to be 167.6 fmol/zone. The glucose assay consisted of spotting increasing concentrations of glucose on the analysis sites and transporting, via capillary action, a solution containing glucose oxidase (GOx), horseradish peroxidase (HRP), and potassium iodide (KI) to the detection sites realizing a yellow-brown color indicating oxidation of iodide to iodine. The device was then dried, scanned, and analyzed to show the correlation between yellow inverse intensity and glucose. Glucose in artificial urine showed good correlation using the devices.

## 1. Introduction

Since the development of the first microfluidic devices (MDs) in the early 1990s, microfluidic technologies have evolved to incorporate a wide range of applications including biomedical analysis, environmental, and agricultural testing, food safety control, medical diagnosis, and drug testing [1]. The most promising real-world application has been point-of-care (POC) diagnostics. POC testing has the potential to greatly improve health care and particularly in resource-limited settings where access to instrumentation, quality medical diagnosis, and proper medication may not always be available [2].

Traditionally, diagnostic tests are performed at central laboratories with well-equipped instrumentation operated by trained personnel. Automation of many more analytical techniques has enabled the analysis of an ever-increasing number of samples and at a lower cost than ever before. Over the past few decades, healthcare has changed dramatically and will continue to do so due to the growing need to provide diagnostic testing and results at the point of care, and because of economic pressures [3]. In addition to home health care needs, POC testing can assist soldiers on the battlefield, populations in rural regions, and in the workplace.

The past decade has seen the development of various substrates to supplant traditional glass and polymeric-based platforms [4,5,6,7,8,9,10,11,12,13,14]. For example, paper as a substrate for microfluidic devices (MDs), first reported by Whitesides et al., has experienced rapid growth in technologies for a myriad of scientific disciplines and industries due to its ease of fabrication, low sample consumption, portability, and low cost [15,16,17,18]. The resultant microfluidic paper-based analytical devices (µPADs) are well documented as platforms for point-of-care (POC) applications and especially in resource-limited settings [19,20,21,22,23,24,25,26,27]. In addition, µPADs have found use in environmental and agricultural testing, food safety control, medical diagnosis, and drug testing [1,15,16,17,18].

Recently, poly nylon thread has gained considerable attention as an inexpensive substrate for POC and biosensor applications [28,29,30,31,32,33,34]. Thread is a useful material to fabricate POC diagnostic devices. For example, aqueous fluids can be easily transported on hydrophilic thread without the use of external forces. Threads are readily available and readily mass produced, are lightweight, can be readily disposed of after use, can be easily functionalized, and manipulated since it can be knitted or woven.

Similar to thread, textiles (e.g., fabric and cloth) have also recently been employed as platforms in MDs [35,36]. The advantages of textiles over paper, for example, include greater tensile and flexibility, variety of fibrous materials, and the ability to form 3D structures [32]. Textiles are also two-dimensional and can be modified to be three-dimensional allowing for a greater diversity of applications unlike thread that is one-dimensional with limited surface area due to small widths and thickness. The benefit of using cord as a platform is than an assay can be done within the cord, without the usage of other material. In some string-based devices, the researches have used paper or fabric for the detection of the reaction and for other purposes. In the light of fabricating simple and cheap microfluidic devices for people in remote areas, cord is a device that requires no other assembly or material to be used effectively.

Typical cord fabrics exist as twisted or rotated yarn woven and fitted with either one or several layers of substrate thereby creating adhesion to rubber mixtures. It can be fabricated from nylon, cotton, polyester, and other materials. Cord can come in a variety of shapes (e.g., lace, ribbon, cable, wire, drawstring, etc.) and is commonly found as long thin flexible string or rope made from several twisted strands. To our knowledge, no study exists that incorporates poly/nylon and cotton cord as a substrate in MDs for enzymatic assays.

Herein, we describe the design and development of µCADs to detect biotinylated goat anti-mouse IgG antibody, rabbit IgG antibody, and glucose. The ELISA and glucose assays employed cotton and poly/nylon cord, respectively. For the ELISA systems, antibody was spotted on the detection site and subjected to a series of reagents and washes. The devices were dried, scanned, and analyzed for intensity (yellow for Strep-ALP or blue for rabbit IgG antibody) correlated to antibody concentration. For the glucose assay, various concentrations of glucose were spotted on the analysis sites and reagents flowed into the region. The devices were then dried, scanned, and analyzed, and the extent of the yellow intensity correlated to the concentration of glucose. It was found that glucose in artificial urine was easily correlated to the µCAD.

## 2. Materials and Methods 

### 2.1. Materials

Streptavidin, alkaline phosphatase conjugate (Strep-ALP) was purchased from Life Technologies (Carlsbad, CA, USA). Trizma base, Tween-20 viscous liquid, alkaline phosphatase yellow (p-NPP) Liquid Substrate System for ELISA, albumin from bovine serum (BSA), a mix-n-stain biotin antibody labeling kit, immunoglobulin G (IgG) from rabbit serum, 5-bromo-4-chloro-3-indolyl phosphate disodium (BCIP), nitrotetrazolium blue chloride (NBT), magnesium chloride, sodium chloride, potassium chloride, sodium dihydrogen phosphate (monobasic), and disodium phosphate (dibasic) were all purchased from Sigma-Aldrich (St. Louis, MO, USA). Affinity-pure goat anti-mouse immunoglobulin G (IgG) was purchased from EMD Millipore Corporation (Burlington, MA, USA). Cord was purchased from Sew Biz Fabrics (Fairlawn, VA, USA). Nitrocellulose membranes (0.45 µm pore) were purchased from Bio-Rad Laboratories (Carlsbad, CA, USA). Scotch packaging tape was purchased from a local bookstore. Glucose oxidase (GOx) (120 U/mL), horseradish peroxidase (HRP) (30 U/mL), sodium acetate trihydrate, sodium dihydrogen phosphate (mono), disodium phosphate (dibasic), potassium iodide, glucose, and acetic acid were purchased from Sigma Aldrich (St. Louis, MO, USA). DS-108 Poly/nylon cord was purchased from Sewbiz Fabrics (Fairlawn, VA, USA).

For the detection of goat anti-mouse IgG antibody, wash and Strep-ALP dilution buffer was 1 M Tris, 0.05% (v/v) Tween (Tris-Tween) (pH = 7.4). Various Strep-ALP solutions (2.50 × 10^−4^–5.00 × 10^−3^ mg/mL) were prepared in the buffer solution. NC was prepared in solution by cutting the NC membrane (5 cm × 3 cm) into smaller square pieces and placing them in chloroform (900 µL) in a closed glass vial. It was then vortexed and allowed to sit for an additional 5 min before spotting onto the detection sites. 1 M Tris 0.05% (w/v) Tween-20 (pH = 7.4) was also prepared by adding HCl to adjust the pH. The goat anti-mouse IgG antibody was labeled with biotin by following instructions in the mix-n-stain biotin antibody labeling kit.

For the detection of rabbit IgG antibody, 0.01 M phosphate buffered saline (pH = 7.4) was prepared and 0.05% (v/v) Tween and 1% (w/v) BSA in phosphate buffer saline (PBS) was also prepared by adding Tween-20 into PBS. Various concentrations of rabbit IgG were made by diluting in PBS (0–67000 fmol/zone). For the secondary antibody, a 1:1000 dilution of the stock ALP-conjugated secondary antibody solution was made in 0.05% (v/v) Tween-20 in PBS. The colorimetric solution for ALP was prepared by preparing a substrate buffer (5 mM MgCl_2_, 100 mM NaCl, 0.05% (v/v) Tween-20 in 100 mM Tris (pH = 9.5)), the substrate buffer was used as the dilution buffer when preparing 5.36 mM BCIP and 3.6 mM of NBT Prior to spotting on the detection site, equal volumes of BCIP and NBT were mixed to create a final concentration of 2.7 mM BCIP and 1.8 mM NBT.

Acetate buffer (0.2 M, pH = 5.1) was prepared by mixing 0.2 M sodium acetate trihydrate (71.5 mL) and 0.2 M acetic acid (28.5 mL). Phosphate buffer (0.1 M, pH = 6) was prepared by mixing 0.2 M sodium dihydrogen phosphate (mono) (87.7 mL) and 0.2 M disodium phosphate (dibasic) (12.3 mL) then diluting the resulting 100 mL of 0.2 M phosphate buffer by making up the volume to 200 mL. GOx was prepared by dissolving 0.0145 g of GOx in 25.0 mL of acetate buffer (0.2 M, pH = 5.1). HRP was prepared by dissolving 0.0048 g in 25.0 mL of phosphate buffer (0.1 M, pH = 6). Potassium iodide (0.6 M) was prepared by dissolving 4.98 g of KI into 50 mL of distilled water. The GOx, HRP, and KI mixture was prepared as follows: solutions of GOx and HRP were added together to obtain a GOx:HRP ratio of 5:1. 500 µL of the GOx/HRP solution is mixed with 500 µL of 0.6 M KI. Various concentrations of glucose (0.0, 0.5, 1.0, 3.0, 4.5, 6.5, 10.0, 12.5 and 15.0 mM) were prepared.

An artificial urine solution was prepared according to the procedure of Brooks and Keevil [34]. In summary, the artificial urine solution was 25 mM ammonium chloride, 2.5 mM calcium chloride, 2.0 mM citric acid, 2.0 mM magnesium sulfate, 25 mM sodium bicarbonate, 90 mM sodium chloride, 10 mM sodium sulfate, 0.4 mM uric acid, 7.0 mM disodium hydrogen phosphate, and 7.0 mM sodium dihydrogen phosphate. The solution was filtered and adjusted to pH = 6 by addition of 1.0 M hydrochloric acid. Varying concentrations of glucose (0.5, 1.0 and 4.5 mM) were added for the study.

### 2.2. Microfluidic Analytical Device Fabrication

#### 2.2.1. μCAD Platform Fabrication

The μCAD used for detection of biotinylated goat anti-mouse IgG was composed of three fabric pads held together by a plastic slip and tape. The inlet was fabric 1 (100% cotton handkerchief, 5 mm × 5 mm), the NC reaction site was fabric 2 (Poly/Nylon Drawstring Cord, 5 mm length × 4 mm width), and the outlet was fabric 3 (100% cotton flannel, 40 mm × 5 mm). Due to varying fabric densities, the types of fabrics were systematically placed so that the arrangement promoted fluid flow via the flannel outlet. NC fabric sites were made in triplicate. Fabric 2 was cut to form the foundation of the three reaction sites and was treated with NC solution (100 μL per site).

**Figure 1:**
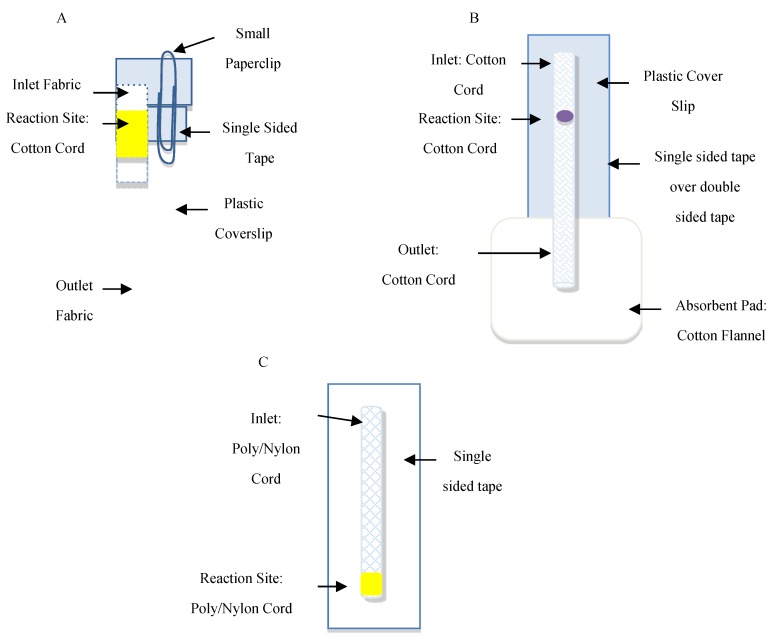
Schematic of μCAD for the detection of (**A**) biotinylated goat anti-mouse IgG, (**B**) rabbit IgG antibodies, and (**C**) glucose.

The NC solution was prepared as described above (Section 2.1) and the NC functionalized piece of cord (site of detection 0.5 × 0.5 cm^2^) was then placed on top of a single-sided tape that was then taped to a plastic cover slip. On the coverslip, the inlet and outlet pieces of fabric were arranged in a manner to help bridge the gap of the detection site to the inlet and outlets, respectively. The open position of the chip allowed for the spotting of reagents onto the cord detection site. The closed position of the chip required a small paperclip and brought all three pieces of materials (cord site of detection, and fabric inlet, and outlet) together for flowing of materials and washing away of unbound materials (Figure 1A). Analysis with Adobe Photoshop CC 2018 obtained inverse yellow color intensity using a fixed sized marquee square tool (6 × 6 mm^2^). The marquee tools allow one to select a shape (rectangular and elliptical), a single row, or column marquee for analysis. The average inverse yellow intensity represents the average control values subtracted from a control sample lacking Strep-ALP enzyme. Table S1 in supporting information shows a summary of the reagents and reaction times for the detection of biotynylated goat anti-mouse IgG for the µCAD platform.

The μCAD for the detection of rabbit IgG antibodies was fabricated from a single piece of 100% cotton cord (60 mm length × ~7 mm width) heat pressed (350 °C for 240 s) to flatten the cord for a smoother surface in which to administer reagents (Figure 1B). For the rabbit IgG system, rabbit IgG (1.5 µL) was spotted on the analysis sites and allowed to dry (10 min). The sites were blocked by adding 1.5 µL of 0.05% (v/v) Tween-20 and 1% (w/v) BSA in PBS, and allowed to dry (10 min). Secondary antibody (1.5 µL) was added and allowed to sit for one min followed by a series of PBS washes (20 × 100 µL) that were flowed through each inlet (wash was flowed 100 µL/min). An absorbent pad (flannel 7 cm × 7 cm) was placed at the end of the device to absorb the wash volume. After the wash, the colorimetric solution (1.5 µL) was spotted and the color-producing reaction was allowed to proceed for 30 min before scanning and analyzing. Analysis with Adobe Photoshop CC 2018, under RGB settings for blue, obtained inverse blue color intensity using a fixed sized marquee circle tool (5 × 5 mm^2^). The average inverse blue intensity represents the average control values subtracted from a control sample lacking rabbit IgG. Table S2 in supporting information shows a summary of the reagents and reaction times for the detection of rabbit IgG antibody for the µCAD platform.

The μCAD device for detection of glucose consisted of a sample site and detection site. The sample and detection sites were situated on opposing ends of the μCAD. The reagents resided in the detection site and the sample solution flowed into the detection site via capillary action.

The μCAD was cut (3 cm length by 0.4 cm width) (Figure 1C). The cord was then heat-pressed (350 °C for 240 s). Varying glucose concentrations were then spotted (5 µL) on the terminal end of the cord and let dry (8 min) before moving on to the enzyme spotting. Alternatively, the reagents (GOx, HRP, and KI) could initially be spotted and then sample flowed into the region. Spotting the glucose first isolated the reagents and product within the detection site, thereby, assisting in the analysis. While the glucose dried, the appropriate enzyme mixtures (GOx, KI, and HRP) in their respective ratios (45 μL), were transported into the micro centrifuge tube. The cords were placed in separate tubes in an upright position with the non-glucose end first. Solutions of enzyme traveled up to the glucose end of the cord via capillary action where reaction occurred. The cords were removed after 10 min, placed flat on a surface, and allowed to dry (30 min). They were scanned and analyzed using a rectangular size marquee tool, 4 × 6 (Adobe Photoshop CC 2018 under CMYK for yellow). To perform the assay using artificial urine, the same procedure was followed with a urine sample.

#### 2.2.2. Choice of Cord

Cord DS-108 was chosen as the material of choice to study biotinylated goat anti-mouse IgG and glucose. It is poly/nylon, is white, and has a number of benefits including low cost, appropriate transport fluid rates, flexibility, easy visualization of fluid transport, and appropriate thickness (1/8 inches) of small volumes. Cord DS-106 was used for the detection of rabbit IgG antibodies. It has similar benefits as the DS-108, but was made of cotton and was 1/4 inches in diameter.

#### 2.2.3. Microfluidic Device Analysis

The chips are scanned with an Epson Perfection V600 Photo Scanner (Suwa, Japan) with a resolution of 1200 dpi. Photoshop CC 2018 was used to determine the mean, mean inverse (yellow and blue) intensities of each analysis site using different size marquee tools. The same size marquee tools were used to analyze the data throughout each different type of assay.

## 3. Results and Discussion

The work described in this paper focused on developing a new microfluidic platform based on poly/nylon drawstring and 100% cotton cord material to quantitatively detect biotinylated goat anti-mouse IgG antibody, rabbit IgG antibody, and glucose via colorimetric analysis.

### 3.1. Detection of Biotinylated Goat Anti-Mouse IgG

**Figure 2:**
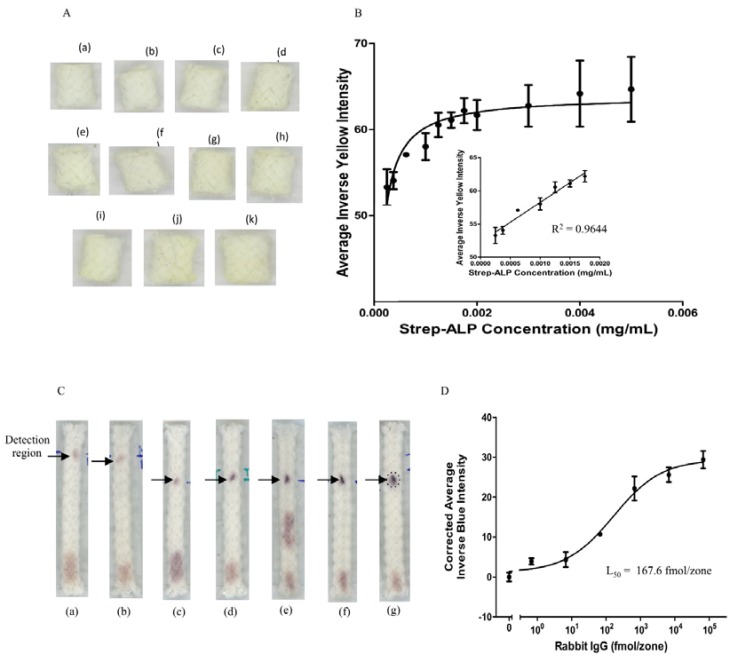
Images of increasing concentrations of (**A**) Strep-ALP: (a) 2.50 × 10^−4^ mg/mL (b) 3.75 × 10^−4^ mg/mL, (c) 6.25 × 10^−4^ mg/mL, (d) 1.00 × 10^−3^ mg/mL, (e) 1.25 × 10^−3^ mg/mL, (f) 1.50 × 10^−3^ mg/mL, (g) 1.75 × 10^−3^ mg/mL, (h) 2.00 × 10^−3^ mg/mL, (i) 3.00 × 10^−3^ mg/mL, (j) 4.00 × 10^−3^ mg/mL, and (k) 5.00 × 10^−3^ mg/mL. (**B**) Inverse yellow mean value intensities as a function of Strep-ALP concentration. Insert is Strep-ALP concentrations ranging from 2.50 × 10^−4^–1.75 × 10^−3^ mg/mL. Data are presented as the average ± SD from three independent measurements. The error bars reflect the standard deviations from the average values. (**C**) Images of increasing rabbit IgG antigen concentrations: (a) 0 fmol/zone, (b) 0.7 fmol/zone, (c) 6.7 fmol/zone, (d) 67 fmol/zone, (e) 670 fmol/zone, (f) 6700 fmol/zone, and (g) 67000 fmol/zone. (**D**) The sigmoidal curve of corrected inverse blue Intensity as a function of rabbit IgG in fmol/zone (L_50_ = 167.6 fmol/zone). Data are presented as the average ± SD from three independent measurements. The error bars reflect the standard deviations from the average values.

To determine the efficiency of the poly/nylon cord-based platform, biotinylated goat anti-mouse IgG was applied to a series of detection sites on the cord, originally pre-spotted with NC, then dried prior to washing with a Tris-Tween solution, followed by increasing concentrations of Strep-ALP, p-NPP, and stop p-NPP solution. After allowing the reaction to proceed for 30 min, a yellow color was observed at the detection sites, indicating completion of the ELISA procedure. The inverse yellow intensity detected is proportional to the concentration of Strep-ALP bound to the biotin labeled antibody found on the detection sites. The images for the μCAD platform are shown in Figure 2A. The variation in yellow color intensity is not large in the Strep-ALP range of concentrations; yet, the scanner is able to differentiate minor differences on increasing concentration. At lower concentrations (2.50 × 10^−4^–1.75 × 10^−3^ mg/mL), a linear range of detection (R^2^ = 0.9644) was observed before it plateaus at the end of the experiment (5.0 × 10^−3^ mg/mL) (Figure 2B). Experiments were performed in triplicate.

The variation in error bars (>10%) may be due to the combined behavior of the materials used for the assay. The inlet and outlet are constructed from 100% cotton, handkerchief, and flannel respectively. The detection site is made from poly/nylon cord and is only slightly hydrophobic before the addition of NC to the site. NC increases the hydrophobicity of the material allowed for enhanced adsorption of biotin labeled IgG protein to the functionalized surface. The enzyme was flowed laterally on the material which and may have been compromised by the fold over piece of tape used to bridge the gap between inlet and outlet fabrics, thus, forming one single piece of textile material.

The graphical representation of the data show that the fabric and cord mixed platform can effectively assess the ELISA system. The cost of manufacturing the μCAD is in the pennies and, because this microfluidic device is reproducible, is worthy of use as a POC device in the healthcare industry especially in resource-limited regions.

### 3.2. Detection of Rabbit IgG Antibodies

Primary rabbit IgG antibody was first spotted 2 cm at the end of the cord and dried. Tween-BSA, ALP-conjugated secondary antibody, and a series of PBS washes were subsequently added followed by the colorimetric substrate for ALP, thereby realizing a blue color after 30 min at the detection sites. The blue color was correlated to the amount of secondary antibody bound to rabbit IgG. The scanned images of the µCAD are shown in Figure 2C and the corrected average blue intensity was plotted as a function of the log of the concentration of rabbit IgG (Figure 2D). The L_50_ was determined to be 167.6 fmol/zone comparable our previous work using a paper ELISA platform (671 ± 208 fmol/zone) [37] and in our recent studies (174 ± 38 fmol/zone) [38]. In this work the lowest amount of Strep-ALP or IgG used was 3.75 × 10^−4^ mg/mL or 0.7 fmol/zone, respectively, which is lower than the limit of detection (LOD) of 4 fmol/zone in a 96-well plate using absorbance detection [37].

The differences observed in the error bars (>10%) may be due to detection site material and size of analysis sites. The cord-based analysis is conducted as a lateral flow assay (LFA) and, because the cord is thick, resembles a three dimensional platform (with multiple weaves of cotton fibers) rather than paper or thread. The size and texture of the cord material, including the many weaves of braided fabric making up the cord, allowed for efficient and sustainable washing away of unbound antibody not bound to antigen on the surface of the cord. The cotton cord was heat pressed which made adsorption of the primary rabbit IgG possible due to the flattened surface as well as removed extra air pockets trapped inside the fibers of the cord.

This μCAD is simple and low cost to fabricate as only small amounts of material (cord, 100% cotton flannel absorbent pad, tape, and paperclip), scissors for cutting, and heat press are required to mass produce and assemble many devices.

### 3.3. Detection of Glucose

To demonstrate the efficiency of the μCAD in a non-ELISA system, we examined a glucose assay. Here, glucose is readily converted to gluconic acid and oxygen reduced to H_2_O_2_ by GOx. H_2_O_2_ is then reduced to water by HRP concomitant with iodide being oxidized to iodine, realizing a yellow-brown color at the detection sites. In a typical experiment, a small aliquot (5 µL) of glucose is spotted at one end of the cord and dried. A solution cocktail of GOx, HRP, and KI is placed in a small tube and the cord is placed vertically such that the solution of cocktail migrates up by capillary action onto the zone of glucose. The cord is let dry for 30 min and scanned.

**Figure 3:**
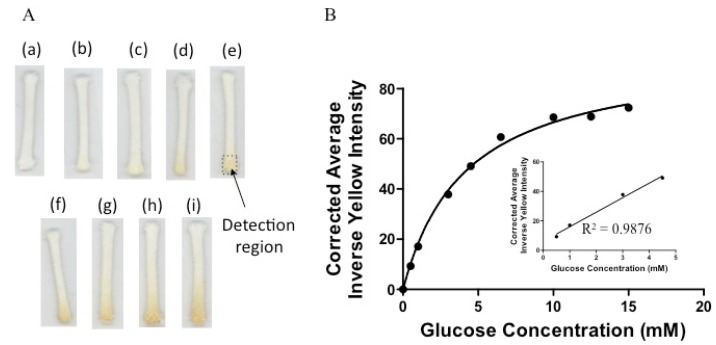
(**A**) Images of increasing concentrations of glucose: (a) 0.00 mM, (b) 0.50 mM, (c) 1.00 mM, (d) 3.00 mM, (e) 4.50 mM, (f) 6.50 mM, (g) 10.0 mM, (h) 12.5 mM, (i) 15.0 mM. (**B**) Corrected average inverse yellow intensities as a function of glucose concentration for the µCAD. Data are presented as the average ± SD from three independent measurements. The error bars reflect the standard deviations from the average values.

Figure 3A shows the images taken of the μCADs for increasing concentrations of glucose. It is apparent that the intensity of the yellow color increases as glucose concentration increases. Figure 3B is a saturation curve of the corrected average inverse yellow as a function of the concentration of glucose. The data and error bars in the figure are the mean and standard deviation, respectively. As can be seen, the responses are linear between 0–4.5 mM glucose and deviate from linearity at higher concentrations. We did not examine concentrations less than 0.5 mM of glucose and did not determine the LOD. Based on our previous work with paper-based devices, we surmise the LOD to be between 0.25–0.50 mM. A glucose concentration of 0.5 mM yields a faint color [2].

The µCAD was examined to measure quantitatively the amount of glucose in artificial urine. Here, known concentrations of glucose (0.5, 1.0 and 4.5 mM) were subjected to a similar procedure and it was found that the device was effective in determining the levels of glucose in several test samples (Table 1) found in healthy and diabetic patients [39,40].

Using the μCAD it is possible to analyze a sample using a simple scanner and with minute quantities of solution. The low material costs of these simple sensors and the ease of their fabrication make them a potential commercial device for developing countries and in remote regions where there is a lack of quality healthcare. The devices can be mass produced in a variety of designs depending on the application. The three percent differences are less than 10%, demonstrating that the calibration curve produced is effective in measuring the glucose concentration of unknown samples.

Cord has several advantages over other commonly used microfluidic-based platform. One, cord is thick and can absorb larger volumes of fluid than paper or thread. Two, cord can function as the external absorbent pad due to its fluidic capacity. Three, cord can be made flat to create a large surface area, reducing the formation of shadows (sometimes found in thread) and simplifying scanning. Four, cord, like thread and paper, does not require external pumps as fluid moves via capillary action.

## 4. Conclusions

We have described the development and application of µCADs in two ELISAs and a glucose assay. Here, the quantitative detection of biotinylated goat anti-mouse IgG antibody, rabbit IgG antibody, and glucose via colorimetric analysis were detailed. Here, antibody and reagent solutions were applied at the detection sites resulting in a change in color that was correlated to the concentration of antibody at the site. Poly/nylon drawstring and 100% cotton cord material is inexpensive, broadly available, easily transported, robust, and has the potential for wash and reuse. Less reagent volume is required than that found in traditional 96-well plate assays, and may be further scaled down by reducing the dimensions of the cord material.

A cord-based platform has potential as a lab-on-a-chip platform as fluids flow along the fabric and cord fibers via capillary action in a lateral flow assay, without the need of external pumps or expensive equipment. Cord may also be an alternative to chromatography paper used in paper microfluidics and polymeric materials like poly(dimethylsiloxane) (PDMS) frequently used in microfluidic devices.

This work demonstrates the viability to detect biomolecules at the femtomolar range, showing high sensitivity using colorimetric substrates. Future work will focus on developing new diagnostic devices for use in the healthcare industry for resource limited settings and developing countries. 

## Figures and Tables

**Table 1:** Comparison of known glucose concentrations to glucose concentrations detected by analysis, with percent difference.

Known Glucose Concentration (mM)	Detected Concentration (mM)	Percent Difference
0.5	0.46 ± 0.05	9.4
1.0	0.92 ± 0.08	8.3
4.5	4.53 ± 0.03	0.7

## Supplementary Materials

The following are available online at https://www.mdpi.com/2072-666X/10/9/614/s1, Table S1: Reagents and reaction wait times for the detection of goat anti-mouse IgG antibody using the µCAD platform as a POC device. Table S2: Reagents and reaction wait times for the detection of rabbit IgG antibody using the µCAD platform as a POC device. Table S3: Reagents and reaction wait times for the detection of glucose using the µCAD platform.
